# Does Palatoplasty in Patients with Cleft Palate Really Improve Otitis Media with Effusion?

**DOI:** 10.3390/dj14020086

**Published:** 2026-02-03

**Authors:** Yosuke Kunitomi, Toshiki Hyodo, Yoshiaki Kitsukawa, Aya Koike, Yasuhiro Tsubura, Yuske Komiyama, Chonji Fukumoto, Takahiro Wakui, Hiroshi Kamioka, Hitoshi Kawamata

**Affiliations:** 1 Department of Oral and Maxillofacial Surgery, Dokkyo Medical University School of Medicine, Tochigi 321-0293, Japan; kunitomi@dokkyomed.ac.jp (Y.K.); hyodo14@dokkyomed.ac.jp (T.H.); y-kitsukawa@dokkyomed.ac.jp (Y.K.); a-koike362@dokkyomed.ac.jp (A.K.); y-tsubura@dokkyomed.ac.jp (Y.T.); y-komi@dokkyomed.ac.jp (Y.K.); chonji-f@dokkyomed.ac.jp (C.F.); 2 Department of Orthodontics, Graduate School of Medicine, Dentistry and Pharmaceutical Sciences, Okayama University, Okayama 700-8525, Japan; kamioka@md.okayama-u.ac.jp; 3 Section of Dentistry, Oral and Maxillofacial Surgery, Sano Kosei General Hospital, Tochigi 327-8511, Japan; 4 Utsunomiya General Service Corps, Japan Ground Self-Defense Forces, Tochigi 321-0145, Japan; 5 Yasu Kazu Charm Dental Clinic, Tochigi 320-0846, Japan

**Keywords:** cleft palate, palatoplasty, otitis media with effusion (OME), velopharyngeal function (VPF)

## Abstract

**Background:** The majority of cleft palate patients have been reported to suffer from otitis media with effusion (OME). The improvement of velopharyngeal function (VPF) after palatoplasty might be evidence for the improvement of the function of the Eustachian tube. The improvement of the function of Eustachian tube by palatoplasty has been reported to be effective for the treatment of OME simultaneously with the insertion of a ventilation tube into the tympanic membrane. There are only a few reports that clearly show the association between improvement of VPF and improvement of OME after palatoplasty. In this study, we discussed whether the improvement of VPF after palatoplasty in cleft palate patients with OME improved OME. **Methods:** Twenty-six patients with cleft palate were included in the study. We retrospectively extracted the information of cleft type, gender, surgical technique, and presence of OME risk factors from electronic medical records. We also investigated the recurrence of OME and the improvement level of VPF at 36 months postoperatively. OME was assessed based on the otolaryngologist’s findings in electronic medical records, with a good prognosis group with no symptom of OME, or a recurrence group with prolonged or recurrent OME. **Results:** At 36 months after palatoplasty, 19 of 23 patients (82.6%) were in the OME good prognosis group and four (17.4%) were in the OME recurrence group. The rate of patients with recurrent OME did not differ significantly by the degree of improvement of VPF. This study indicated that clear association between other risk factors for OME and OME recurrence could not be shown. **Conclusion:** We observed that most patients with cleft palate who underwent palatoplasty showed a good prognosis for OME at 36 months after surgery. However, further studies are needed to investigate the impact of different surgical techniques on the improvement of OME and the degree to which VPF improves, as well as the effect of each OME risk factor.

## 1. Introduction

Cleft lip and/or palate is a facial deformity that occurs in approximately 1 in 500 live births in Japan. The incidence of cleft lip and/or palate in Japan is higher than that in other countries [1]. It has been reported that more than 96% of cleft palate patients suffer from otitis media with effusion (OME). Abnormal attachment and abnormal function of the tensor veli palatini muscle and levator veli palatini muscle in cleft palate patients affects the function of the Eustachian tube [2]. OME is one of the causes of hearing impairment [3] and hearing impairment has a significant impact on the language development of pediatric patients. Early treatment for OME is therefore important for patients with cleft palate. The treatment of OME in patients with cleft palate requires the improvement of the function of the Eustachian tube. The improvement of velopharyngeal function (VPF), in other words, the improvement of the function of the tensor veli palatini muscle and levator veli palatini muscle after palatoplasty, might be evidence for the improvement of the function of the Eustachian tube. Until the improvement of the function of the Eustachian tube, the insertion of a ventilation tube into the tympanic membrane performed simultaneously with palatoplasty has been reported to be effective for the treatment of OME [4]. We usually perform palatoplasty at the age of 18 months in patients with cleft palate, and at the same time, otolaryngology–head and neck surgeons also perform the insertion of a tympanic membrane ventilation tube in cleft palate patients with OME. Saranyoo et al. reported the recurrence rate and risk factors for OME in cleft palate patients after palatoplasty [2]. Iemura-Kashiwagi et al. reported the effectiveness of tympanic ventilation tube insertion during palatoplasty in patients with OME [4]. Furthermore, Yoshitomi et al. reported the association of the width of cleft palate with the incidence of OME [5]. However, there are only a few reports that clearly show the association between the improvement of VPF and the improvement of OME after palatoplasty [6]. According to the cited systematic review, they reported the independent relation of the surgical procedure for palatoplasty with the recurrence of OME, the indications for tympanic membrane ventilation tubes, and VPF. However, there is no mention of interrelationships between surgical procedures for palatoplasty and the recurrence of OME and VPF. In this study, we investigated the recurrence of OME at 36 months after palatoplasty in cleft palate patients with OME, and we also examined VPF (speech–language assessment and blowing test) and other risk factors for OME prevalence in cleft palate patients. Then, we discussed whether the improvement of VPF after palatoplasty in cleft palate patients with OME really improved OME.

## 2. Patients, Materials and Methods

### 2.1. Patients

Twenty-six patients with cleft palate who were treated in our department, the Department of Oral and Maxillofacial Surgery, Dokkyo Medical University Hospital, between January 2014 and April 2021 were included in the study. Patients with symptomatic CLP and submucous cleft palate were excluded. All of the patients underwent palatoplasty in our department and the insertion of a tympanic membrane ventilation tube by otolaryngology–head and neck surgeons simultaneously. Palatoplasty was performed using the Furlow or Pushback technique, depending on the condition of cleft palate based on a single surgeon’s decision. We retrospectively extracted the information of cleft type, gender, surgical technique (Furlow or Pushback), and the presence of OME risk factors (gender, adenoid hypertrophy, allergic rhinitis, family history of otitis media) [7,8,9,10,11] from the electronic medical record. Three patients discontinued follow-up visits during the postoperative observation period, preventing evaluation of VPF and OME. Therefore, OME evaluation was performed on 23 patients. All patients are in the pre-orthodontic treatment stage and have not undergone orthodontic treatment. The parents of the patients were fully informed about the surgical procedures and written informed consent was obtained from all of them.

### 2.2. Assessment of VPF

VPF was assessed at 36 months after surgery. This was when the children were around 4.5 years of age and when the assessment of speech was possible. VPF was examined separately in the Furlow technique group and the Pushback technique group. VPF was assessed by a speech–language assessment and the leakage of exhaled air from the nostrils (blowing test). Quantitative evaluation for VPF would be ideal; however, our hospital does not have the systems in place to perform nasometry or acoustic nasalance measurements, so we do not perform them. Furthermore, endoscopic examinations are performed only in a limited number of cases due to the age and cooperation level of pediatric patients. According to the Japan Society of Logopedics and Phoniatrics, speech–language assessment and the blowing test are recommended as simple, qualitative methods with good reproducibility for evaluating VPF in patients with CLP [12]. Therefore, we adopted these assessment methods in this study. For speech–language assessment, we used the protocol reported by Miura et al. [13], and a speech–language–hearing therapist (ST), who was certified as a specialist for dysphagia rehabilitation from the Japanese Association of Dysphagia Rehabilitation, tested for the presence of hypernasality and consonant distortion according to the protocol [13]. The protocol also measures the presence of nasal snorts, along with the degree of expiratory nasal leakage. Consonant distortion due to hypernasality and expiratory nasal leakage is evaluated on a four-point scale: none, mild, moderate, and severe. Nasal noise is evaluated on a two-point scale: present or absent. The degree of expiratory nasal leakage is evaluated using a nasal speculum on a three-point scale: none (−), 0–2 cm (+), and more than 2 cm (++). Leakage of exhaled air from the nostrils (blowing test) was assessed by ST using a nasal speculum. According to the degree of nasal leakage of the exhaled air, the leakage was assessed as none (−), 0–2 cm (+) and more than 2 cm (++). As an overall assessment of VPF from the speech–language assessment and the leakage of exhaled air from nostrils, the patients were classified as ‘Severe (velopharyngeal insufficiency in the assessments)’, ‘Mild (any one of the abnormalities in the assessments)’, ‘None (no abnormality in the assessments)’.

### 2.3. Evaluation of OME Recurrence

We investigated the recurrence rate of OME and the status of tympanic ventilation tube placement (Retained, Removed or Dropped) at 36 months after surgery. Symptoms of OME were assessed based on otoscopic findings by otolaryngologists on electronic medical records. The otoscopic examination was not quantitative. Then, we simply evaluated the symptoms of OME as a good prognosis group with no symptoms of OME, or a recurrence group with prolonged or recurrent OME.

### 2.4. Statistical Analysis

Fisher’s exact test was used to compare OME recurrence rates between groups. Univariate and multivariate analyses were performed using gender, type of palatoplasty procedure, degree of VPF, presence or absence of adenoid hypertrophy, presence or absence of allergic rhinitis, family history of OME, and cleft type as explanatory variables. Statistical analysis was performed using IBM SPSS Statistics Ver. 29, and *p* value < 0.05 was considered significant for each test.

### 2.5. Ethics

The research protocol was approved by the ethics committee of Dokkyo Medical University School of Medicine (R-81-12J, on 14 April 2024), and the protocol was provided in the outpatient office and on the webpage of the department.

## 3. Results

### 3.1. Classification of Cleft Type and Gender

The cleft type was classified as cleft lip and palate (cleft extending from lip and alveolus to hard palate and soft palate) in nine of 26 cases (one bilateral, eight unilateral), cleft of hard palate and soft palate (cleft extending from most of the hard palate to the soft palate) in 11 cases, cleft of soft palate with minor posterior cleft of hard palate (cleft restricted from the posterior edge of the hard palate to soft palate) in three cases and cleft of soft palate in three cases (Table 1).

### 3.2. Surgical Technique for Palatoplasty

**Figure 1:**
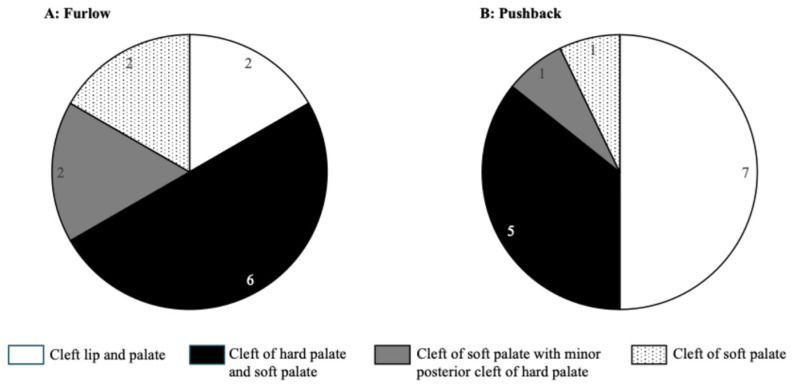
Surgical technique for palatoplasty. (**A**) The Furlow technique was performed in 12 patients. (**B**) The Pushback technique was performed in 14 patients.

The Furlow technique was used in 12 patients, including two with cleft lip and palate, six with cleft hard palate and soft palate, two with cleft of soft palate with minor posterior cleft of hard palate, and two with cleft of soft palate. The Pushback technique was used in 14 patients, including seven with cleft lip and palate, five with cleft hard palate and soft palate, one with cleft of soft palate with minor posterior cleft of hard palate, and one with cleft of soft palate (Figure 1). The surgical method of the palatoplasty was decided by the surgeon, depending on the characteristics of individual patients.

### 3.3. OME Recurrence Rate

At 36 months after palatoplasty, 19 of 23 patients (82.6%) were in the OME good prognosis group and four (17.4%) were in the OME recurrence group. Eight of 11 patients (72.7%) were in the OME good prognosis group and three (27.3%) were in the OME recurrence group for the Furlow technique. Eleven of 12 patients (91.7%) were in the OME good prognosis group and one (8.3%) was in the OME recurrence group for the Pushback technique. There was no significant difference in OME recurrence rates between the two surgical techniques.

### 3.4. Status of Tympanic Ventilation Tubes at 36 Months After Surgery

**Figure 2:**
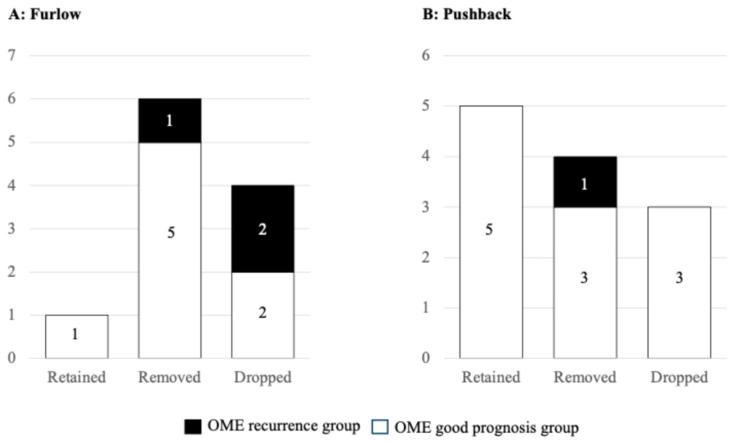
Status of tympanic ventilation tubes at 36 months after surgery. (**A**) In the Furlow technique group, OME symptoms were found in three patients (27.2%, one patient in removed group, two patients in dropped group). (**B**) In the Pushback technique group, OME symptoms were found in one patient (8.3%, one patient in removed group).

In 23 patients, the status of tympanic ventilation tubes was examined at 36 months after surgery. The average placement period of tympanic ventilation tube was 21 months for the Furlow technique group and 25 months for the Pushback technique group. At 36 months after surgery, the tympanic ventilation tube was retained in one of 11 patients (9.0%) in the Furlow technique group; the patient had undergone re-tubing (Figure 2A). In the Pushback technique group, the tympanic ventilation tube was retained in five of 12 patients (41.6%); two of five tube-retained patients had undergone re-tubing (Figure 2B). The otolaryngologist removed the tube in six of 11 patients (54.6%) in the Furlow technique group and in four of 12 patients (33.4%) in the Pushback technique group, because there might be no findings of OME. The tube dropped out but was not re-tubed in four of 11 (36.4%) patients in the Furlow technique group and three of 12 cases (25.0%) in the Pushback technique group. At 36 months after palatoplasty, the otolaryngologist found OME symptoms in three patients (27.2%, one patient in removed group, two patients in dropped group) in the Furlow technique group, and in one patient (8.3%, one patient in removed group) in the Pushback technique group (Figure 2A,B).

### 3.5. Relationship of VPF with OME Recurrence at 36 Months After Surgery

**Figure 3:**
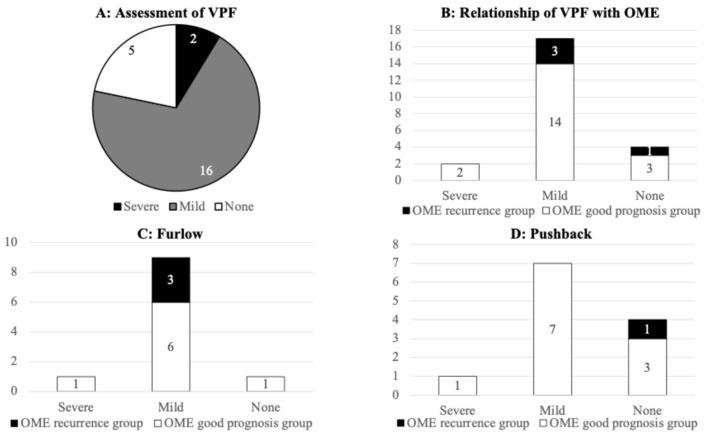
Relationship of VPF with OME recurrence at 36 months after surgery. (**A**) VPF was assessed as ‘Severe’ in two cases, ‘Mild’ in 16 cases, and ‘None’ in five cases. (**B**) The number of patients with OME recurrence was zero (0%), three (17.6%), and one (25%) in the ‘Severe,’ ‘Mild,’ and ‘None’ category of VPF, respectively. (**C**) In the Furlow technique group, OME recurrence were observed with VPF assessment of ‘Mild’. (**D**) In the Pushback technique group, OME recurrence was observed with VPF assessment of ‘None’.

VPF was assessed as ‘Severe (velopharyngeal insufficiency)’ in two cases, ‘Mild (any of the abnormalities)’ in 16 cases and ‘None (no abnormality)’ in five cases (Figure 3A). The number of patients with OME recurrence was zero (0%), three (17.6%), and one (25%) in the ‘Severe,’ ‘Mild,’ and ‘None’ category of VPF, respectively. The rate of patients with OME recurrence did not differ significantly by the degree of improvement of VPF (Figure 3B). In the Furlow technique group, the VPF assessment was ‘Severe’ in one case, ‘Mild’ in nine cases, and ‘None’ in one case. In the Pushback technique group, the VPF assessment was ‘Severe’ in one case, ‘Mild’ in seven cases, and ‘None’ in four cases (Figure 3C,D). In the Furlow technique group, three patients with OME recurrence were observed with VPF assessment of ‘Mild’, and in the Pushback technique group, one patient with OME recurrence was observed with VPF assessment of ‘None’ (Figure 3C,D).

### 3.6. Association Between OME Recurrence and General Risk Factors

**Figure 4:**
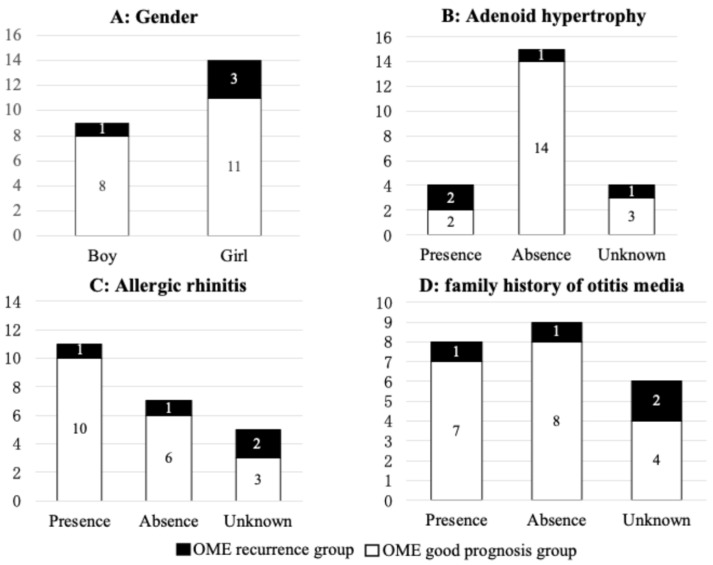
Association between OME recurrence and general risk factors. (**A**) Gender. (**B**) Adenoid hypertrophy. (**C**) Allergic rhinitis. (**D**) Family history of otitis media.

With regard to general risk factors for OME, one out of nine boys and three out of 14 girls showed OME recurrence, with no significant difference between the genders (Figure 4A). OME recurrence was observed in two out of four patients with adenoid hypertrophy and one out of 15 patients without adenoid hypertrophy, but with no significant difference (*p* = 0.097) (Figure 4B). OME recurrence was observed in one of 11 patients with allergic rhinitis and one of seven patients without allergic rhinitis, with no significant difference (Figure 4C). OME recurrence was observed in one out of eight cases with a family history of otitis media and in one out of nine cases without a family history of otitis media, with no significant difference (Figure 4D). We performed univariate and multivariate analyses to examine the impact of local risk factors and general risk factors for recurrence of OME (Table S1). However, no significant difference was observed for any factor. Therefore, the cause in the few OME recurrence cases remains unclear.

## 4. Discussion

In the present study, 19 of 23 patients (82.6%) showed good prognosis of OME at 36 months after palatoplasty. However, since we did not compare the prognosis of OME in the patients who underwent palatoplasty with the prognosis in untreated cleft palate patients of the same age, it was difficult to say whether surgical intervention really improved OME. We could not find significant differences in the degree of improvement of VPF and OME recurrence rate at 36 months after palatoplasty. One reason for this is that only non-invasive examinations could be performed in the evaluation of VPF due to the patient’s age. Furthermore, in some cases, even the non-invasive examinations did not provide sufficient cooperation, making accurate evaluation impossible. Current examinations for the Eustachian tube function are invasive [14], and it might be difficult to apply these invasive examinations to children. Therefore, non-invasive examinations for the Eustachian tube function for children are expected to be developed. Another reason for this is that since all patients underwent simultaneous insertion of ventilation tubes, the favorable course of OME cannot be attributed exclusively to palatoplasty. Furthermore, we should recognize that the lack of statistically significant associations between VPF, surgical technique, and OME recurrence should also be interpreted within the limits of the statistics.

Allergic rhinitis and adenoid hypertrophy have also been reported as risk factors for OME in children [7,8,9,10,11]. In the present study, a clear association between these risk factors and OME recurrence could not be shown. As we proceed with the treatment of cleft palate, we need to keep in mind factors other than VPF that are involved in the development and/or recurrence of OME.

The ideal surgical technique for palatoplasty is that the surgery does not inhibit maxillary bone development and provides the adequate acquisition of VPF. The Pushback technique, where full thickness muco-periosteal and muco-muscular flaps are used for closing the cleft, allows enough posterior extension of the soft palate, but postoperative inhibition of maxillary bone development has been noted [15,16,17]. The Furlow technique, where muco-muscular flaps with a double opposing Z-plasty on the soft palate are used for closing the cleft, is less invasive to the hard palate and has less inhibition on maxillary bone development, but it has been reported that it is difficult to apply to cases with a wide cleft [18]. In this study, the Furlow technique was the first choice for palatoplasty and the Pushback technique was chosen for cases with a wide cleft, based on a surgeon’s decision. The choice of surgical techniques based on the surgeon’s decision according to the severity of cleft palate introduces a significant bias in statistical analyses regarding the relationship with VPE and the risk of recurrence of OME. This constitutes a potential confounding factor that could not be fully controlled in this study. However, this study was not primarily designed to evaluate the relative merits of surgical techniques for improving VPF or OME. According to the systematic review [6], five papers examined the relationship between palatoplasty techniques and OME recurrence, with four reporting no association between the surgical approach and OME recurrence. This finding was consistent with our study results. Furthermore, the systematic review [6] reported the independent relation of the surgical procedure for palatoplasty with the recurrence of OME, the indications for tympanic membrane ventilation tubes, and VPF. However, there is no mention of interrelationships between surgical procedures for palatoplasty, recurrence of OME, and VPF. In this study, only one severe case of velopharyngeal insufficiency was observed in both the Pushback technique and the Furlow technique. This indicates that both surgical techniques were performed appropriately. It was clearly demonstrated that VPF improved in most cases, resulting in an improvement in OME. We performed univariate and multivariate analyses using surgical techniques as explanatory variables for the recurrence of OME. As a result, the surgical technique was not an independent risk factor for OME recurrence. In our hospital, cases of severe VPF or recurrent OME after palatoplasty in patients with CLP were also infrequent. Studies investigating risk factors for poor prognosis cases would require a substantial number of cases.

In general, the reason why OME is common in patients with cleft palate is that abnormal attachment and abnormal function of the tensor veli palatini muscle and levator veli palatini muscle in cleft palate patients affects the function of the Eustachian tube. Therefore, it is believed that restoration of the function of the tensor veli palatini muscle and levator veli palatini muscle through palatoplasty, and improvement of Eustachian tube function through palatoplasty, contribute to improvement of OME [19,20]. During analysis, patients whose ventilation tubes remained in place but showed no recurrence or relapse of OME may indicate that, despite the absence of improvement in Eustachian tube function, the presence of the tubes simply prevented OME relapse. We discussed this point with otolaryngologists, who told us that when OME had improved in the patients, the tubes were left in place until they fell out, considering the specific nature of CLP in these children. Therefore, in this study, patients whose tympanic membrane ventilation tubes remained in place, and in whom OME was absent, were also evaluated as belonging to the good prognosis group. However, it is crucial to examine whether OME recurs after removal of tympanostomy tubes in these cases. We are continuing to make our observations in collaboration with otolaryngologists. It is important to establish clear evidence by conducting not only a single-center study but also a multi-center collaboration study. A multi-center prospective study with standardized VPF assessment and objective otologic endpoints (tympanometry, audiometry) would be valuable.

## 5. Conclusions

We observed that most patients with cleft palate who underwent palatoplasty showed a good prognosis for OME at 36 months after surgery. However, further studies are needed to investigate the impact of different surgical techniques on the improvement of OME and the degree to which VPF improves, as well as the effect of each OME risk factor.

## Figures and Tables

**Table 1:** Classification of cleft type and gender.

Cleft Type			Boy	Girl	Total
Cleft lip and palate	Unilateral	Left	2	1	3
		Right	3	2	5
	Bilateral		1	0	1
	Subtotal		6	3	9
Cleft of hard palate and soft palate			3	8	11
Cleft of soft palate with			0	3	3
minor posterior cleft of hard palate					
Cleft of soft palate			1	2	3
	Subtotal		4	13	17
Total			10	16	26

## Data Availability

The original contributions presented in this study are included in the article/supplementary material. Further inquiries can be directed to the corresponding author.

## Supplementary Materials

The following supporting information can be downloaded at: https://www.mdpi.com/article/10.3390/dj14020086/s1, Table S1. Factors associated with the OME recurrence.

## Abbreviations

The following abbreviations are used in this manuscript:

OME	Otitis Media with Effusion
VPF	Velopharyngeal Function
