# Supplementary material for: Does Palatoplasty in Patients with Cleft Palate Really Improve Otitis Media with Effusion?
Source: Dent J (Basel). 2026 Feb 3;14(2):86. doi: 10.3390/dj14020086 (PMC12939578; doi:10.3390/dj14020086)
Supplement: Supplementary file 1 [file dentistry-14-00086-s001.zip › dentistry-3951249-supplementary.pdf]

**Table S1.** Factors associated with the OME recurrence.

| Variable | Univariate Analysis | | | Multivariate Analysis | | |
|---|---|---|---|---|---|---|
| | OR | 95% CI | *p*-Value | OR | 95% CI | *p*-Value |
| **Gender** | | | | | | |
| Females VS Males | 0.39 | 0.04-4.26 | 0.443 | 0,17 | 0.004-7.73 | 0.363 |
| **Surgical Technique** | | | | | | |
| Pushback VS Furlow | 1.88 | 0.25-14.0 | 0.541 | 0.38 | 0.02-6.77 | 0.509 |
| **VPF** | | | | | | |
| Mild, None VS Severe | 0.00 | 0.00-0.00 | 0.999 | 0.00 | 0.00-0.00 | 0.999 |
| **Adenoid hypertrophy** | | | | | | |
| Absence VS Presence | 1.73 | 0.52-5.74 | 0.373 | 3.16 | 0.31-32.29 | 0.332 |
| **Allergic rhinitis** | | | | | | |
| Absence VS Presence | 1.24 | 0.31-4.94 | 0.760 | 2.81 | 0.16-49.88 | 0.48 |
| **Family history of OME** | | | | | | |
| Absence VS Presence | 1.06 | 0.29-3.92 | 0.931 | 0.74 | 0.13-4.32 | 0.734 |
| **Cleft type** | | | | | | |
| Soft VS Hard | 0.30 | 0.03-2.65 | 0.278 | 0.06 | 0.001-2,72 | 0.147 |

Abbreviations: CI = Confidence Interval, OR = Odds Ratio.
